# Characterizing the Clinical and Genetic Spectrum of Polycystic Ovary Syndrome in Electronic Health Records

**DOI:** 10.1210/clinem/dgaa675

**Published:** 2020-09-22

**Authors:** Ky’Era V Actkins, Kritika Singh, Donald Hucks, Digna R Velez Edwards, Melinda Aldrich, Jeeyeon Cha, Melissa Wellons, Lea K Davis

**Affiliations:** 1 Department of Microbiology, Immunology, and Physiology, Meharry Medical College, Nashville, Tennessee; 2 Vanderbilt Genetics Institute, Vanderbilt University Medical Center, Nashville, Tennessee; 3 Division of Genetic Medicine, Department of Medicine, Vanderbilt University Medical Center, Nashville, Tennessee; 4 Vanderbilt Epidemiology Center, Institute of Medicine and Public Health, Vanderbilt University Medical Center, Nashville, Tennessee; 5 Division of Quantitative Sciences, Department of Obstetrics and Gynecology, Vanderbilt University Medical Center, Nashville, Tennessee; 6 Department of Thoracic Surgery, Vanderbilt University Medical Center, Nashville, Tennessee; 7 Department of Biomedical Informatics, Vanderbilt University Medical Center, Nashville, Tennessee; 8 Division of Diabetes, Endocrinology, and Metabolism, Department of Medicine, Vanderbilt University Medical Center, Nashville, Tennessee

**Keywords:** polycystic ovary syndrome, phenotyping, hormones, electronic health record, polygenic risk scores

## Abstract

**Context:**

Polycystic ovary syndrome (PCOS) is one of the leading causes of infertility, yet current diagnostic criteria are ineffective at identifying patients whose symptoms reside outside strict diagnostic criteria. As a result, PCOS is underdiagnosed and its etiology is poorly understood.

**Objective:**

We aim to characterize the phenotypic spectrum of PCOS clinical features within and across racial and ethnic groups.

**Methods:**

We developed a strictly defined PCOS algorithm (PCOS_keyword-strict_) using the International Classification of Diseases, ninth and tenth revisions and keywords mined from clinical notes in electronic health records (EHRs) data. We then systematically relaxed the inclusion criteria to evaluate the change in epidemiological and genetic associations resulting in 3 subsequent algorithms (PCOS_coded-broad_, PCOS_coded-strict_, and PCOS_keyword-broad_). We evaluated the performance of each phenotyping approach and characterized prominent clinical features observed in racially and ethnically diverse PCOS patients.

**Results:**

The best performance came from the PCOS_coded-strict_ algorithm, with a positive predictive value of 98%. Individuals classified as cases by this algorithm had significantly higher body mass index (BMI), insulin levels, free testosterone values, and genetic risk scores for PCOS, compared to controls. Median BMI was higher in African American females with PCOS compared to White and Hispanic females with PCOS.

**Conclusions:**

PCOS symptoms are observed across a severity spectrum that parallels the continuous genetic liability to PCOS in the general population. Racial and ethnic group differences exist in PCOS symptomology and metabolic health across different phenotyping strategies.

Polycystic ovary syndrome (PCOS) is an endocrine disorder that is the leading cause of infertility in females. The genetic, environmental, and metabolic variables that contribute to its complex architecture also influence clinical heterogeneity among individuals with PCOS, resulting in a broad spectrum of symptoms. The heterogeneous clinical presentation of PCOS makes it difficult to diagnose, and an estimated 75% of females with PCOS remain undiagnosed (1, 2).

There are 3 commonly used diagnostic criteria for PCOS that each have specific symptom requirements for diagnosis. The first diagnostic definition was put forth by the National Institutes of Health (NIH), which required an ovulatory phenotype (eg, oligomenorrhoea) and hyperandrogenism for diagnosis (3, 4). Subsequently, the second diagnostic definition for PCOS was created by the European Society for Human Reproduction and Embryology and the American Society for Reproductive Medicine, commonly referred to as the *Rotterdam criteria*. The Rotterdam diagnostic criteria were designed to expand the PCOS definition from the strict NIH definition by requiring any 2 of the following 3 symptoms: oligoanovulation, hyperandrogenism, or polycystic ovaries (3, 5). However, the Androgen Excess and PCOS Society argued that hyperandrogenism is the primary driver of PCOS and thus, created a third diagnostic criteria that requires both hyperandrogenism and ovulatory dysfunction (eg, oligoanovulation or polycystic ovaries) (4). PCOS diagnostic criteria were developed to improve and standardize diagnoses; however, patients identified by each approach may have differing symptomologies and only a small fraction of females will meet multiple diagnostic criteria (6, 7).

Implementing a broader PCOS definition could lead to false-positive diagnoses and prevalence overestimations (4, 8). However, restrictive definitions miss females with clinical manifestations outside the requirements (9), including metabolic dysfunction. Females with PCOS and metabolic dysfunction are at higher risk for a range of comorbidities, including insulin resistance, type 2 diabetes, cardiovascular disease, obesity, cancer, and psychiatric disorders (10, 11). Delayed screening and treatment for PCOS could exacerbate these metabolic conditions, particularly in underrepresented populations that have a greater baseline risk of metabolic disorders (12, 13). Studies have shown that African American and Hispanic females with PCOS have an increased risk for metabolic syndrome and other cardiovascular disorders, yet little is known about why comorbidities increase across diverse racial and ethnic groups and whether expanding diagnostic criteria for PCOS may improve health equity (14, 15).

Most prior studies included small numbers of minority females and many have used differing diagnostic criteria (1, 16-18). Without larger studies, it is difficult to accurately characterize the clinical presentation of PCOS and its consequences across populations. Adapting an inclusive phenotyping approach capable of identifying severe to mild PCOS presentations could improve study methods for marginalized communities.

The overarching hypothesis of this study is that clinically diagnosable PCOS represents the extreme tail (ie, clinical manifestation) of a spectrum of hormonal and metabolic dysregulation that has common manifestations in a large number of females. In our study, we developed a simple but stringent automated phenotyping algorithm that could be applied to electronic health records (EHRs) to identify females with confirmed PCOS. Next, we systematically relaxed the inclusion criteria to test the hypothesis that excess androgens, increased metabolic dysfunction, and PCOS genetic liability are present to a lesser degree among females with milder PCOS symptoms and symptoms outside the diagnostic criteria. Finally, we examined these features across diverse racial and ethnic groups within the EHR.

## Materials and Methods

### Clinical data

Vanderbilt University Medical Center (VUMC) is a tertiary care hospital in Nashville, Tennessee, with several clinics throughout Tennessee and the surrounding states. Medical records have been electronically documented at VUMC since the early 1990s (19). The VUMC EHR houses more than 3 million EHRs in a deidentified clinical research database called the Synthetic Derivative (SD). The synthetic derivative includes demographics, International Classification of Diseases, ninth revision (ICD9) and tenth revision (ICD10) codes, procedural codes, clinical notes, medications, and laboratory values. The Vanderbilt University Institutional Review Board (IRB) approved this project (IRB 160 279).

### Algorithm development

**Figure 1.**
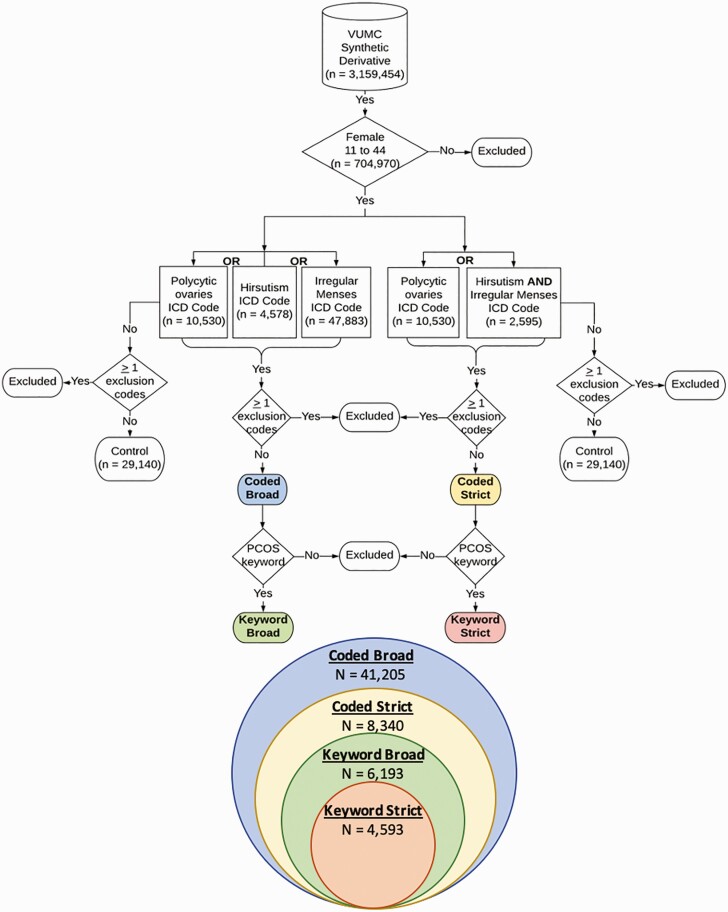
Polycystic ovary syndrome (PCOS) algorithm pipeline. This schematic illustrates the algorithm development steps for the controls and the 4 PCOS algorithms. The Venn diagram illustrates how the algorithm data sets are nested within one another. Each color corresponds with one of the algorithm data sets. ICD, International Classification of Diseases, 9th and 10th revision.

We identified females with PCOS within the synthetic derivative using a strict automated phenotyping algorithm that yielded a positive predictive value (PPV) of at least 90%. Our secondary analysis relaxed the requirements of our core algorithm to identify a larger sample of patients with a wider array of PCOS symptoms. In total, we developed 4 algorithms, which are illustrated in Fig. 1. The demographics of each resultant data set are reported in Table 1.

**Table 1.** Polycystic ovary syndrome (PCOS) algorithm descriptive statistics of women identified as PCOS cases and controls

	Controls	Coded broad	Coded strict	Keyword broad	Keyword strict
No.	29 140	41 205	8340	6193	4593
NH White	22 569	25 977	5418	4154	3101
NH African American	3278	7555	1195	887	648
Hispanic	1421	2053	368	274	204
HIS White	1014	1462	264	203	154
HIS African American	33	69	13	11	8
Average age, y (SD)	23.85 (9.47)	24.96 (7.97)	25.18 (7.63)	24.71 (7.52)	25.09 (7.39)
Average EHR record length (SD)	3.92 (3.9)	4.52 (4.54)	4.23 (4.15)	4.69 (4.22)	4.66 (4.13)
Medical home, %	65	64	60	66	66
Average clinical values (SD)					
Body mass index	25.71 (7.43)	28.61 (8.44)	34.12 (9.10)	33.50 (9.14)	34.54 (9.07)
Insulin, mcU/mL	20.08 (18.99)	24.86 (22.83)	27.17 (23.05)	25.34 (21.94)	27.27 (21.58)
Estradiol, pg/mL	82.22 (69.03)	89.92 (69.74)	75.92 (61.13)	71.64 (56.56)	75.57 (60.49)
Free testosterone, pg/mL	3.58 (2.45)	4.70 (2.94)	5.70 (3.23)	5.41 (3.01)	5.73 (3.09)

Abbreviations: EHR, electronic health record; HIS, Hispanic; NH, non-Hispanic.

Before applying each algorithm, we filtered the entire synthetic derivative population to include only females between the ages of 11 to 44 years to enrich our population for females of reproductive age. We first deployed the PCOS_keyword-strict_ algorithm, which required at least one ICD9 or ICD10 code for polycystic ovaries (ie, 256.4 and E28.2) OR hirsutism (ie, 704.1 and L68.0) AND irregular menses (ie, all 626 codes, all N91 codes, N92.5, and N92.6) (Supplementary Table 1) (20). Hirsutism was used as clinical evidence of hyperandrogenism. Individuals with any exclusion codes (Supplementary Table 1) (20) present in their medical record were removed. Furthermore, the PCOS_keyword-strict_ algorithm required the presence of at least one of the following keywords: “polycystic ovaries,” “PCOS,” or “PCO” in a clinical note. We included inpatient and outpatient note types designated as “outpatient note,” “gynecology clinic visit,” “nursing report,” “endocrine and diabetes clinic visit,” “pediatric endocrinology patient visit,” and “reproductive endocrinology clinic visit.” Next, to broaden the approach, we dropped the keyword requirements and retained only the ICD codes for the PCOS_coded-strict_ algorithm. We then loosened the requirement of the union of hirsutism and irregular menses codes and instead required the presence of ICD codes for polycystic ovaries OR hirsutism OR irregular menses. In addition to any code for these conditions, we required the presence of a PCOS-related keyword. The resultant algorithm was termed *PCOS*_*keyword-broad*_. Last, we again dropped the keyword requirement, which resulted in the broadest approach (PCOS_coded-broad_), including the presence of any PCOS symptoms (ie, ICD codes for polycystic ovaries OR hirsutism OR irregular menses).

To identify controls, the synthetic derivative was again filtered for females between the ages of 11 to 44 years. The control data set excluded individuals with one or more exclusion ICD codes or any inclusion codes used to define PCOS case criteria (Supplementary Table 1) (20).

### Chart review methods for algorithm evaluation

We performed 2 comprehensive chart reviews to evaluate the performance of the phenotyping algorithms. The charts were reviewed by clinical domain experts (M.W. and J.C.) and trained reviewers (K.V.A., D.H., and K.S.). We first evaluated the PPV for each algorithm. We randomly selected 50 charts from each set of algorithm-defined cases (200 in total) for manual chart review by trained reviewers and clinical domain experts (21). We then calculated the proportion of true positive cases based on manual chart review out of the total algorithm identified cases, to define the PPV for each algorithm (Table 2).

**Table 2.** Prevalence of polycystic ovary syndrome (PCOS) algorithms and chart review results. Prevalence of PCOS was calculated for everyone identified in each algorithm (overall) and for White, African American, and Hispanic women identified in each algorithm. Chart review was conducted on 50 random records identified by each PCOS algorithm (n = 200), and algorithm performance was measured by positive predictive value

	Coded broad	Coded strict	Keyword broad	Keyword strict
Overall				
No.	41 205	8340	6193	4593
EHR prevalence, %	5.8	1.2	0.88	0.65
PPV, %	30	98	82	96
White				
No.	26 317	5526	4207	3143
EHR prevalence, %	6.6	1.4	1.1	0.79
African American				
No.	7648	1217	899	658
EHR prevalence, %	9.5	1.5	1.1	0.81
Hispanic				
No.	2052	368	274	204
EHR prevalence, %	6.7	1.2	0.90	0.67

Abbreviations: EHR, electronic health record; PPV, positive predictive value.

Next, we evaluated the sensitivity and specificity of the original PCOS_keyword-strict_ algorithm. For this review, we first identified a “medical home” population (defined by the presence of at least 5 ICD codes on separate days over at least a 3-year period [N = 752 436]). Then, we filtered for females who met a data floor requirement (ie, at least one ICD10 code for PCOS, E28.2 [N = 9507]). From this sample, we then selected a new set of 200 charts at random and performed a manual chart review of all available information in the EHRs (ie, codes and clinical notes) to classify individuals into “PCOS cases” and “not PCOS cases” based on clinical gold standards for diagnosis. This manual review clinical determination was then compared to the PCOS_keyword-strict_ algorithm-defined determination to calculate sensitivity and specificity (Table 3).

**Table 3.** Sensitivity and specificity for polycystic ovary syndrome algorithm data sets. A 2 × 2 contingency table showing the positive predictive value, negative predictive value, sensitivity, and specificity of the keyword strict (KS) algorithm. The KS algorithm was compared to the gold-standard definition. Chart review was conducted on 200 random records of women who met our data floor and medical home definition

		Keyword strict algorithm		
		Case	Not case	Results
Gold Standard	Case	96	97	50% Sensitivity
	Not case	5	2	29% Specificity
	Results	95% PPV	2% NPV	

Abbreviations: NPV, negative predictive value; PPV, positive predictive value.

### Laboratory data

Weight and height measurements are routinely recorded during clinical visits and were extracted from the EHRs to calculate body mass index (BMI). Laboratory measurements were extracted and cleaned using QualityLab (22). In brief, 3075 quantitative laboratory tests were extracted from EHRs of 70 704 patients in the synthetic derivative. Laboratory values with nonnumeric values (n = 2865) and those evaluated on only one patient (n = 467) were removed. The remaining laboratory values were cleaned by filtering out duplicate entries and measurements with multiple units. Insulin, estradiol, and free testosterone hormone data were then extracted for further analysis. Median laboratory values were calculated for individuals with multiple clinical measurements. Median laboratory values of BMI, insulin, estradiol, and free testosterone were used to characterize the clinical features of each algorithm dataset (Table 4 and Supplementary Table 2) (20). For each laboratory value, extreme outliers (ie, first and 99th percentile) were removed. Additionally, laboratory values were regressed on age at the laboratory and an inverse normal transformation was applied to the residual to result in an age-adjusted normalized laboratory value for subsequent genetic analyses (Supplementary Tables 4 and 5) (20). Sample sizes differed for subsequent analyses based on available clinical measurements (Supplementary Fig. 1) (20).

**Table 4.** Laboratory measurements for polycystic ovary syndrome algorithm cases and controls. Wilcoxon rank sum test was performed between cases and controls. The 25th and 75th percentile was reported for each algorithm

Algorithm	Type	No.	Median	25th Percentile	75th Percentile	*P*	
	Body mass index						
Coded broad	Cases	34 603	26.54	22.21	33.50	< 2.22E-16	^*b*^
	Controls	29 140	24.03	20.68	29.30		
Coded strict	Cases	7555	33.605	27.28	40.36	< 2.22E-16	^*b*^
	Controls	29 140	24.03	20.68	29.30		
Keyword broad	Cases	5844	32.805	26.29	39.70	< 2.22E-16	^*b*^
	Controls	29 140	24.03	20.68	29.30		
Keyword strict	Cases	4358	34.08	27.63	40.76	< 2.22E-16	^*b*^
	Controls	29 140	24.03	20.68	29.30		
	Insulin						
Coded broad	Cases	257	17.4	10.00	24.10	.0001	^*b*^
	Controls	14 475	13.65	5.50	20.90		
Coded strict	Cases	150	19.05	11.20	28.70	1.50E-06	^*b*^
	Controls	14 475	13.65	5.50	20.90		
Keyword broad	Cases	134	18.1	10.52	26.06	.00088	^*a*^
	Controls	14 475	13.65	5.50	20.90		
Keyword strict	Cases	109	19.4	11.53	32.53	2.40E-05	^*b*^
	Controls	14 475	13.65	5.50	20.90		
	Estradiol						
Coded broad	Cases	196	63.5	38.00	116.00	.0081	
	Controls	18 799	55.5	32.00	108.00		
Coded strict	Cases	67	54	38.00	96.65	.79	
	Controls	18 799	55.5	32.00	108.00		
Keyword broad	Cases	58	54.5	38.50	90.88	.86	
	Controls	18 799	55.5	32.00	108.00		
Keyword strict	Cases	48	55.5	39.50	93.20	.64	
	Controls	18 799	55.5	32.00	108.00		
	Free testosterone						
Coded broad	Cases	159	4.4	2.25	6.58	.0016	^*a*^
	Controls	125	3	1.70	4.95		
Coded strict	Cases	75	5.9	3.00	7.70	3.80E-06	^*b*^
	Controls	125	3	1.70	4.95		
Keyword broad	Cases	73	5.2	2.90	7.40	3.50E-05	^*b*^
	Controls	125	3	1.70	4.95		
Keyword strict	Cases	53	5.6	3.60	7.50	1.40E-05	^*b*^
	Controls	125	3	1.70	4.95		

^*a*^ *P* < .002.

^*b*^ *P* < 4 × 10^–3^.

### Genetic data

We included 40 802 females of European descent and 9418 females of African descent who were genotyped on the Illumina MEGA^EX^ Array (23). There were 1 832 777 genotyped variants pre–quality control and preimputation. Full details of the quality control pipeline have been described in Dennis et al (22). In summary, we removed single-nucleotide variations (SNVs, formerly single-nucleotide polymorphisms [SNPs]) with a call rate of less than 0.98, individuals with a call rate of less than 0.98, and individuals with a discrepancy between chromosomal and reported sex. FlashPCA2 was used for principal component analysis (24, 25). Related samples with a pi-hat greater than 0.2 (approximate first-cousin relationships) were removed. The Michigan Imputation server was used to impute missing genotypes based on the Haplotype Reference Consortium panel and imputation accuracy was set at *R*^2^ greater than 0.03 (26). For the European genotyped sample, 36 604 696 variants were included in the final data set and 36 513 773 variants were included in the African genotyped sample.

### Construction of polycystic ovary syndrome polygenic risk scores

Polygenic risk scores (PRS) for PCOS were calculated for each individual using the PRS-CS software package, which applies a Bayesian continuous shrinkage model to SNV β estimates to adjust for linkage disequilibrium (27). With this framework, no *P* value thresholds are required to generate PRS. β estimates were drawn from the summary statistics of the largest meta–genome-wide association study (GWAS) of PCOS performed by Day and colleagues that included 5209 cases and 32 055 controls of European descent diagnosed using NIH or Rotterdam criteria (28). To maximize our ability to assess the full polygenicity of PCOS, all SNVs (n_European_ = 783 989, n_African_ = 772 724) from the summary statistics data set were used to generate PCOS_PRS._ PCOS_PRS_ were calculated for a total of 40 802 females of European descent and 9418 females of African descent in BioVU.

### Statistical analysis

The prevalence of each algorithm phenotype was calculated using the proportion of cases identified by each algorithm out of the total number of females in the synthetic derivative between ages 11 to 44 years (see Table 2). The PPV was determined by calculating the proportion of “reviewer-defined” true positives out of the “algorithm-defined” positives (see Table 2). Sensitivity was defined as the number of “algorithm-defined” positives out of the total positives (ie, “algorithm-defined” positives plus “reviewer-defined” positives). Specificity was defined as the number of “algorithm-defined” negatives out of the total negatives (ie, “algorithm-defined” and “reviewer-defined” negatives) (Table 3).

**Figure 2.**
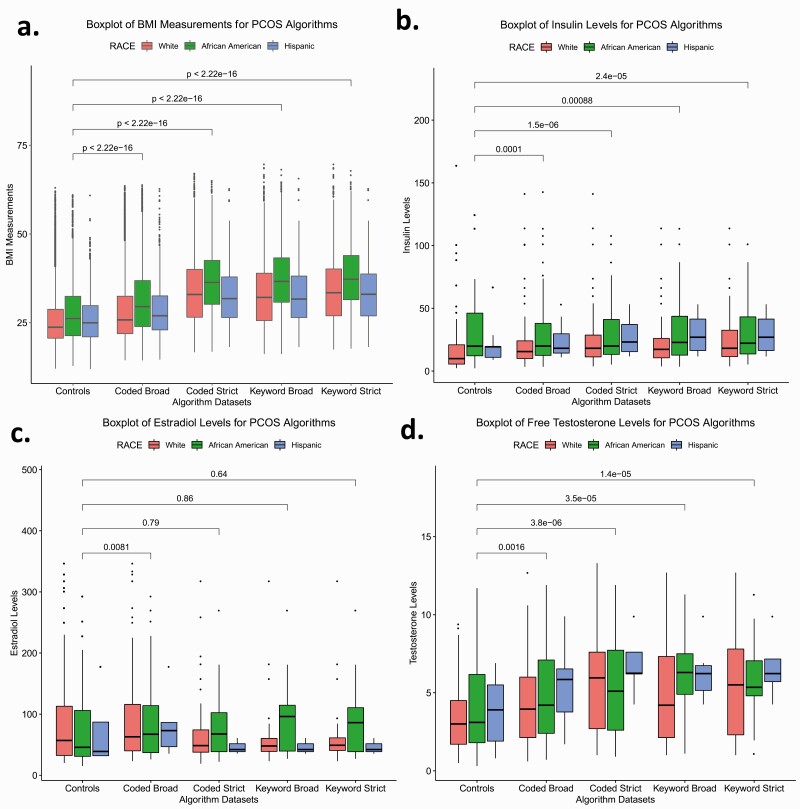
Laboratory measurements for polycystic ovary syndrome (PCOS) algorithm cases and controls. Box plots of A, body mass index (BMI) measurements; B, insulin levels; C, estradiol levels; and D, free testosterone levels for PCOS algorithm cases and controls. Colored boxes represent races. Boxes represent the individuals with lab measurements in the 25th and 75th percentile. Lines above and below the boxes represent the 95th and 5th quartiles. Lines within each box mark the median. Wilcoxon rank sum tests were performed between algorithm cases and controls and brackets display the *P* values of the statistical test.

**Figure 3.**
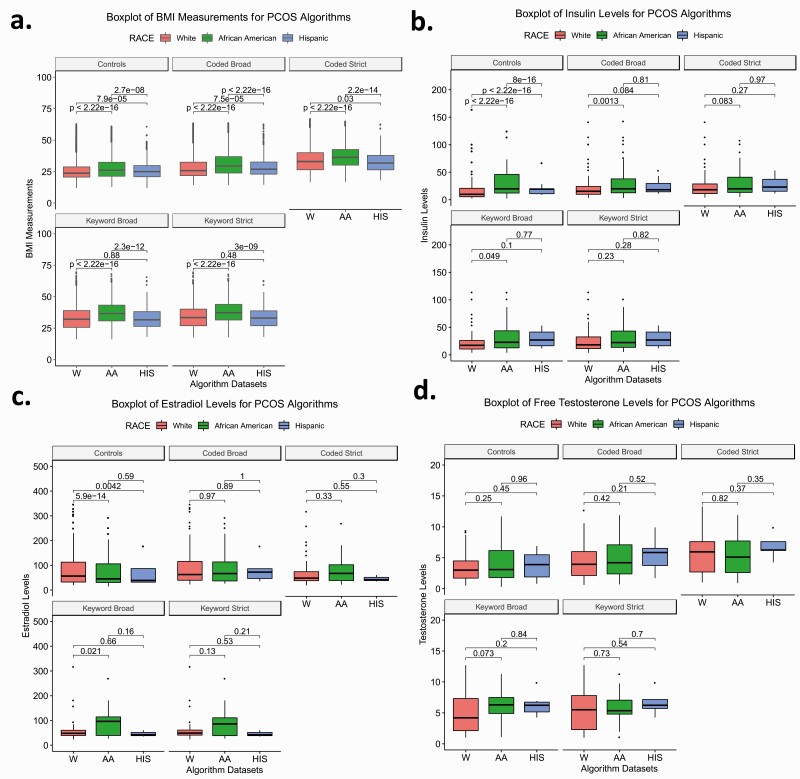
Comparison of laboratory measurements between White (W), African American (AA), and Hispanic (HIS) polycystic ovary syndrome (PCOS) algorithm cases and controls. Box plots of A, body mass index (BMI) measurements; B, insulin levels; C, estradiol levels; and D, free testosterone levels for W, AA, and HIS PCOS cases and controls. Colors correspond with each race. Boxes represent the 25th and 75th quartiles. Lines above and below the boxes represent the 95th and fifth quartiles. Lines within each box mark the median. Wilcoxon rank sum tests were performed between races within the PCOS cases and control data sets. Brackets display the *P* values of the statistical test.

**Figure 4.**
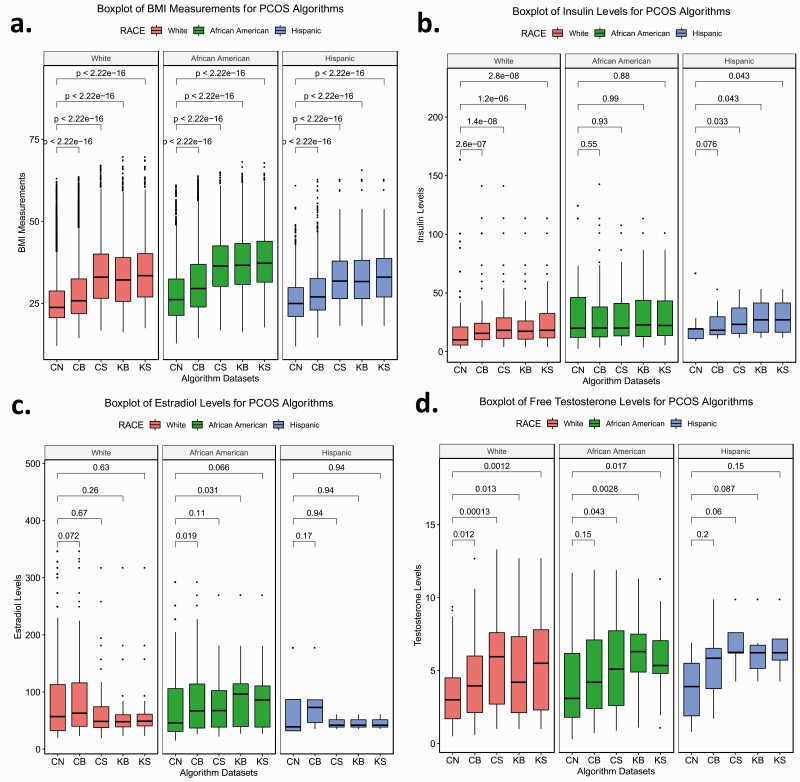
Race-stratified laboratory measurements for polycystic ovary syndrome (PCOS) algorithm cases and controls. Box plots of A, body mass index (BMI) measurements; B, insulin levels; C, estradiol levels; and D, free testosterone levels of race stratified PCOS algorithm data sets. Each color corresponds with a race. Boxes represent the 25th and 75th quartiles. Lines above and below the boxes represent the 95th and fifth quartiles. Lines within each box mark the median. Wilcoxon rank sum tests were performed between algorithm cases and controls within each race. Brackets display the *P* values of the statistical test. CB, coded broad; CN, controls; CS, coded strict; RB, keyword broad; RS, keyword strict.

We performed Wilcoxon rank sum tests to determine if the median BMI and laboratory measurements (eg, free testosterone, insulin, and estradiol) differed between the PCOS algorithm-identified cases and controls. We then tested the difference between cases defined by each algorithm and between cases and controls stratified by EHR-reported race. Finally, we evaluated differences between African American, White, and Hispanic females identified as cases by each algorithm (Table 4, Supplementary Table 2, Figs. 2-4, Supplementary Figs. 2 and 3) (20).

We performed logistic and linear regression analyses to test the association between PCOS_PRS_ and case/control designation and, separately, the association between PCOS_PRS_ and available hormone data (ie, estradiol, free testosterone, insulin). The analysis was performed in 2 populations based on genetic ancestry: European descent (n = 8925) and African descent (n = 3381). Analyses were filtered to individuals who met our medical home definition. Covariates included median age (calculated across the medical record for each individual) and the top 10 genetic principal components to adjust for genetic ancestry when fitting the case/control logistic regression model. A separate sensitivity analysis adjusting BMI was also performed (Supplementary Table 3) (20). For regression analysis of hormone data, covariates included the top 10 genetic principal components. BMI was included as a covariate in the sensitivity analysis. We then evaluated the model and variance explained by PCOS_PRS_ using the Nagelkerke pseudo-*R*^2^ for the logistic regressions and *R*^2^ for the linear regressions (Table 5, Supplementary Tables 3-5) (28). All analyses were performed in R 3.6.0.

**Table 5.** Genetic validation of polycystic ovary syndrome (PCOS) diagnosis. Logistic regressions were performed between PCOS case status and PCOS polygenic risk scores (PCOS_PRS_). Regressions were adjusted for the first 10 principal components, and each algorithm data set was case matched for age and race

Algorithm	Cases	Controls	Estimate	SE	*P*	OR	LCI	UCI	PRS *R*^2^
European									
Coded broad	1877	5052	0.06	0.03	.06	1.06	1.00	1.13	7.00E-04
Coded strict	366	5052	0.17	0.06	.01	1.18	1.05	1.33	0.004
Keyword broad	332	5052	0.25	0.06	6.97E-05	1.28	1.14	1.45	0.008^*a*^
Keyword strict	245	5052	0.20	0.07	.01	1.22	1.06	1.41	0.005
African									
Coded broad	908	1663	0.11	0.06	.07	1.12	0.99	1.26	0.002
Coded strict	161	1663	0.34	0.12	.01	1.40	1.11	1.78	0.01
Keyword broad	129	1663	0.30	0.13	.03	1.34	1.03	1.75	0.007
Keyword strict	98	1663	0.40	0.15	.01	1.49	1.11	2.01	0.011

Abbreviations: LCI, lower confidence interval; OR, odds ratio; PRS, polygenic risk score; *R*^2^, pseudo-R^2^; UCI, upper CI.

^*a*^ *P* < .002.

We included 2 corrections for multiple testing. The first corrected threshold reflects adjustment for all independent tests (n = 30, *P* < .002, indicated by ^*a*^ in tables). The second corrected threshold is more stringent and reflects adjustment for all tests (n = 120, *P* < 4 × 10^–3^, indicated by ^*b*^ in tables) regardless of whether they were independent or performed on nested subsets of the data (see Fig. 1).

## Results

### Sample characteristics

PCOS data set demographics are reported in Table 1. Potential PCOS cases were selected from 704 970 females between ages 11 to 44 years in the synthetic derivative (see Fig. 1). Among them, 399 405 females had an EHR-reported race of White, 80 892 females reported as African American, and 30 445 females reported as Hispanic. The remaining females had a different EHR-reported race, with the largest majority being unknown. Although the sample sizes differed between the algorithms, each algorithm identified a similar proportion of positive cases across racial/ethnic groups. The most stringent algorithm, PCOS_keyword-strict_, identified 4593 potential PCOS cases including 3143 White, 658 African American, and 204 Hispanic females (see Table 2). As the algorithm requirements were relaxed, the number of individuals meeting symptom criteria increased. PCOS_keyword-broad_ identified 6193 individuals including 4207 White, 899 African American, and 274 Hispanic females. The PCOS_coded-strict_ approach identified 8340 individuals including 5526 White, 1217 African American, and 368 Hispanic females. At the broadest extreme, the PCOS_coded-broad_ (n = 41 205) algorithm captured the largest and most diverse sample, with 26 317 White, 7648 African American, and 2052 Hispanic females.

The individuals identified by the keyword algorithms had the highest average medical record length at 4.69 years (SD = 4.22) for PCOS_keyword-broad_ and 4.66 years (SD = 4.13) for PCOS_keyword-strict_. This was expected because the increased length of the record also increased the probability of PCOS documentation in the medical record. The PCOS_coded-strict_ and PCOS_keyword-strict_ algorithms captured the eldest set of patients who were in their mid-20s with a median age of 25.18 (SD = 7.63) and 25.09 (SD = 7.39) years, respectively. Comparatively, the control data set had the youngest sample, aged 23.85 (SD = 9.47) years with the shortest EHR length at 3.92 (SD = 3.9). As expected, the control sample had the lowest average clinical measurements for BMI, insulin, estradiol, and free testosterone (see Table 1).

### Evaluation of algorithm performance

We found little difference between the PPVs calculated for the PCOS_keyword-strict_ algorithm using 2 distinct chart review methods. PCOS_keyword-strict_ yielded a PPV of 96% using our first method of chart review (see Table 2) and a PPV of 95% when it was tested against the clinician gold standard definition in our second manual review method (see Table 3). Despite this, the PCOS_keyword-strict_ algorithm had a sensitivity of only 50% and specificity of 29% when evaluated using the gold-standard definition (see Table 3).

When compared to our broader algorithms, the performance of the PCOS_keyword-strict_ algorithm was most similar to the PCOS_coded-strict_ algorithm, which had a slightly higher PPV of 98% (see Table 2). The text mining criteria did not significantly improve the accuracy of PCOS_keyword-strict_ over PCOS_coded-strict_. The accuracy of the “broad” criteria decreased compared to the “strict” algorithms. Our broadest algorithm, PCOS_coded-broad_, had the lowest performance, with a PPV of 30% (see Table 2). This is likely due to the large number of patients who had only an irregular menstruation ICD code with no other diagnosed PCOS symptoms. Interestingly, the addition of the text-mined PCOS keyword significantly improved the PPV of the broad criteria algorithm from 30% (PCOS_coded-broad_) to 82% (PCOS_keyword-broad_). Unlike the extreme broad criteria, the other algorithm phenotypes were similarly prevalent (18, 29, 30). This was also true for the race-stratified cohorts, for which there were little to no differences in prevalence across algorithm phenotypes.

### Clinical feature characterization

We characterized the clinical profiles of cases and controls identified by each algorithm by calculating median BMI, insulin, estradiol, and free testosterone levels (see Table 4). Each algorithm identified set was significantly heavier compared to controls (*P* < .05). For all algorithms, African American and Hispanic females had higher baseline metabolic profiles for BMI, insulin, estradiol, and free testosterone measurements compared to White females. Moreover, the median BMI in the algorithm data sets increased with increasing algorithm stringency (Fig. 2A). African American females meeting the algorithm criteria consistently demonstrated the highest median BMI in the PCOS_coded-broad_, PCOS_coded-strict_, and PCOS_keyword-broad_ algorithms (Supplementary Table 2) (20). The BMI of all 3 stratified race groups differed significantly from each other with the exception of White and Hispanic females in the strictest keyword algorithms (Fig. 3A). Within the Hispanic-defined group, the median values did not vary significantly between the PCOS_coded-strict_, PCOS_keyword-broad_, and PCOS_keyword-strict_ groups (Supplementary Fig. 3A) (20).

Overall, females identified by each of the 4 PCOS algorithms demonstrated significantly higher insulin and free testosterone levels compared to controls (*P* < .05) (Fig. 2B and 2D, Table 4). The median hormone values also increased with algorithm stringency, though the trend was not as pronounced as it was for the BMI measurements. BMI increased with algorithm stringency within each race stratified analysis. Insulin values increased with algorithm stringency for White and Hispanic females, and free testosterone values increased with algorithm stringency for White and African American females (Fig. 4, Supplementary Table 2) (20).

Measurements of free testosterone were significantly higher among females identified by the PCOS_coded-strict_, PCOS_keyword-broad_, and PCOS_keyword-strict_ algorithms compared to the controls and the PCOS_coded-broad_ algorithm (Supplementary Fig. 2D) (20). The insulin profile of African Americans was similar across females identified by each algorithm and also did not significantly differ from controls (Fig. 4B). However, African American females identified by the PCOS_coded-broad_ and PCOS_keyword-broad_ algorithms did exhibit higher estradiol levels than African American controls (Supplementary Table 2, Fig. 4C) (20). Additionally, African American females identified by the PCOS_keyword-broad_ algorithm demonstrated significantly higher estradiol levels than White females identified by the same approach (Fig. 3C). Outside of these findings, there were no other significant differences between races for hormone values (see Fig. 3).

### Genetic validation of polycystic ovary syndrome case status

**Figure 5.**
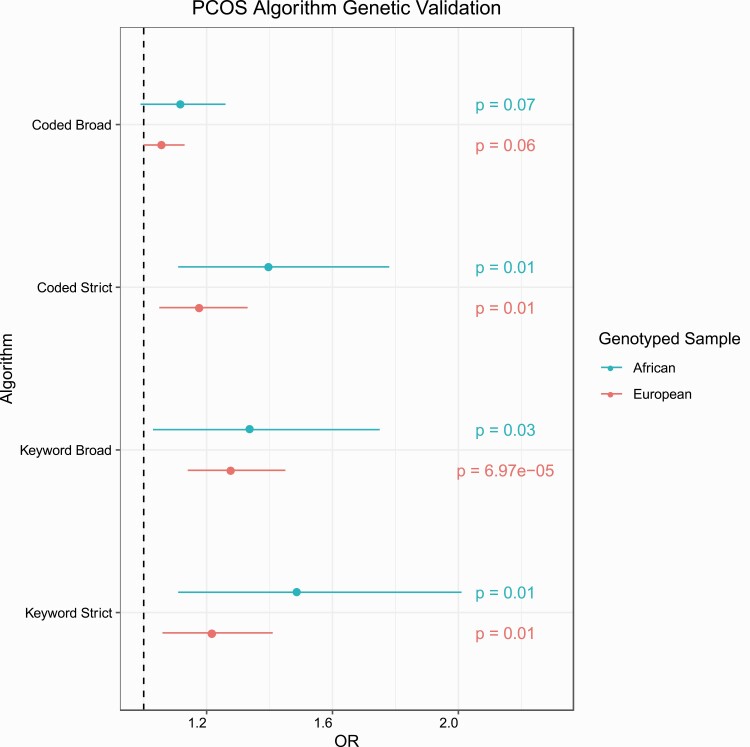
Genetic validation of polycystic ovary syndrome (PCOS) algorithms. Logistic regressions were performed between PCOS case status and PCOS polygenic risk scores (PCOS_PRS_). The regression models were adjusted for median age and the first 10 principal components. *P* values are displayed for each regression model. Colors correspond to the genotyped sample used in the analysis. OR, odds ratio.

Increasing algorithm requirement stringency was also reflected in the performance of the PCOS_PRS_. Odds ratios (ORs), which are reported per synthetic derivative of the PCOS_PRS_, were higher among cases identified by algorithms using strict PCOS requirements and decreased as those requirements were relaxed. This trend was observed both for the European and African genotyped samples (Fig. 5, Table 5). BMI was tested as a covariate in the model and did not impact the results. (Supplementary Table 3) (20).

Among females identified as cases and controls in the European descent PCOS_keyword-broad_ algorithm, we observed a significant association between PCOS_PRS_ and case/control status (see Fig. 5 and Table 5). Cases identified by the PCOS_keyword-broad_ algorithm yielded the highest OR of 1.28 (95% CI, 1.14-1.45, *P* = 6.97E-05) and a PRS pseudo-*R*^2^ of 0.8%. Comparatively, the PCOS_coded-strict_ algorithm yielded the second highest OR of 1.22 (95% CI, 1.06-1.41, *P* = .01) with a PRS pseudo-*R*^2^ of 0.5%.

Although none of the models for the African-descent genotyped samples passed our multiple correction thresholds, the PCOS_coded-strict_, PCOS_keyword-broad_, and the PCOS_keyword-strict_ algorithms passed a false discovery rate of 0.05 and demonstrated higher point estimates compared to the European sample. The African descent PCOS_keyword-strict_ algorithm yielded the highest OR (OR = 1.49, 95% CI, 1.11-2.01, *P* = .01) and had the greatest variance explained in case/control status by PCOS_PRS_ model (PRS pseudo-*R*^2^ = 1.1%). This was followed by the PCOS_coded-strict_ algorithm with an OR of 1.40 (95% CI, 1.11-1.78, *P* = .01) and then, the PCOS_keyword-broad_ algorithm with an OR of 1.34 (95% CI, 1.03-1.75, *P* = .03).

### Association between polycystic ovary syndrome polygenic risk scores and hormone measurements

There were no significant associations between PCOS_PRS_ and the hormone measurements (insulin, estradiol, and free testosterone) evaluated because of the large CIs. However, PCOS_PRS_ explained 11% of the phenotypic variance for estradiol in the African-descent PCOS_coded-strict_ cases and controls (Supplementary Table 4) (20). The highest *R*^2^ observed among European-descent females was in estradiol levels measured in the PCOS_coded-strict_ algorithm sample.

## Discussion

Expanding study inclusion criteria has the potential to increase and diversify future study samples, which may significantly benefit underrepresented high-risk groups (29). This study describes an automated PCOS phenotyping method that captures varying degrees of PCOS symptoms, parallel dysregulated hormone levels, and increased genetic risk for the PCOS_coded-strict_, PCOS_keyword-broad_, and PCOS_keyword-strict_ algorithms. Our results also further support the hypothesis that females with PCOS symptoms have increased metabolic dysregulation compared to controls when measured on BMI, insulin, and free testosterone values dependent on clinical definitions and their EHR-reported race. Finally, our study demonstrates the importance of including African-descent genotyped samples, in addition to European-descent populations, for genetic analysis.

Our 2 “strict definition” algorithms identified PCOS cases with high accuracy (PPV > 95%), and the PCOS_keyword-strict_ algorithm performed just as well when compared to the gold-standard data set (PPV = 95%). Although the sensitivity and the specificity of the PCOS_keyword-strict_ algorithm was low, the high PPV displays its high accuracy within an EHR setting, suggesting that these algorithms are not likely to be useful for clinical purposes but are useful for identifying PCOS study populations. Compared to the previously reported ICD-based PCOS algorithm, which had a PPV of 66% and 65% for the “broad” and “refined” algorithms, our 2 best performing algorithms improved case identification by more than 30% (9). The Castro et al (9) study and our study both show the feasibility of using ICD-based algorithms to identify PCOS cases and the advantages of using EHR systems. Consistent with the previous study, we also found that the inclusion of text mining increased the proportion of true PCOS cases (PPV = 98%) but resulted in a smaller sample size. Removal of the text mining requirement only slightly affected the PPV (96%), indicating that studies performed in EHR systems with limited access to clinical notes and ultrasound images may still be able to identify females with PCOS with high accuracy. Conversely, we found that the addition of text mining to the broad algorithms significantly improved the accuracy from 30% to 82%, indicating that clinical suspicions of PCOS documented in medical records may also be used to identify females with PCOS even when few symptoms are coded.

The clinical and epidemiological features of our PCOS cohorts are consistent with previous studies that show females with PCOS have higher metabolic clinical characteristics than controls (31-33). We also observed no significant difference in the prevalence of PCOS definitions between minority and White individuals, nor were there any large differences between races for insulin, estradiol, and free testosterone clinical measurements outside of higher estradiol levels in African Americans in the PCOS_coded-strict_ algorithm (34). This finding could illustrate an increase in aromatization caused by higher circulating androgen levels or may simply be a reflection of increased ovulatory activity observed among African American females (33, 35, 36). However, this proves difficult to definitively conclude because of the small sample size, the large variability of the data in the cases and controls, and the timing at which estradiol was measured, which could have been at any point during the individual’s menstrual cycle. It is also important to note that African American females had significantly higher BMI measurements than Hispanic and White females across both the control and PCOS definition data sets. African Americans are disproportionately affected by conditions such as obesity and type 2 diabetes, which could possibly be exacerbated by the onset of PCOS (37, 38). These conditions could also be influenced by the patient structure of the EHR database, which has a sicker population because of the tertiary care status of VUMC and its geographic location, where obesity rates are generally higher.

The spectrum of PCOS characteristics remain the root of its heavily debated diagnostic criteria, and PRS provided a unique way to validate the phenotypes identified by our algorithms outside the traditional clinical symptomology. PCOS has a complex genetic architecture evidenced by its high heritability (~ 70%) and its underlying polygenic background (39, 40). PCOS_PRS_ exploits this by assessing the cumulative SNV-based risk across different clinical definitions and ancestries. Although it is ideal to calculate PRS using summary statistics obtained from population references that accurately portray our respective target group, to date there are no PCOS GWAS in African-descent populations. Thus, we were limited to using summary statistics from a European GWAS to build PRS both for our European- and African-descent samples, even though it is known that European-based PRS do not perform as robustly in non-European samples (41). Despite this, previous studies have shown that PCOS_PRS_ models can detect cross-ancestry genetic risk in African and multiancestry cohorts using European-based PRS models (42). In our study, the PCOS_PRS_ model explained little variance in case/control status (PRS pseudo-*R*^2^ ≤ 1%), but the PCOS_PRS_ was significantly higher among cases in the PCOS_coded-strict_, PCOS_keyword-broad_, and the PCOS_keyword-strict_ algorithms compared to controls.

The limitations of our study include lower statistical power in the genetic analysis of African-descent cases and controls. This limitation is exacerbated by the underrepresentation of non-European populations in the PCOS GWAS, but also reflects the low representation of non-European samples in the VUMC EHRs. Eurocentric GWAS remain a significant limitation to interpretability of PRS in non-European samples because it is unrepresentative of the genetic diversity that make up those populations. Therefore, there is a high need for studies to increase the genetic data gathered on diverse ancestral populations to advance PCOS genomics in EHRs (41, 43).

In conclusion, we applied an automated phenotyping approach to examine variation in the symptomology of PCOS across racial and ethnic groups using both strict and broad classification criteria. Debate over current implemented PCOS diagnostic criteria, in part, reflects the complexity of PCOS symptoms, and EHRs provide significant advantages. First, research in the longitudinal EHR allows us to maximize the number of PCOS clinical manifestations observed over time (16, 30, 44). Second, EHRs contain a variety of clinical variables (laboratory measurements, imaging records, etc) that can be used to study the risk factors and health outcomes of PCOS patients. We created a simple EHR-based PCOS algorithm that identifies patients using ICD codes and text mining (PCOS_keyword-strict_) methods, and by relaxing the inclusion requirements we identified patients across the PCOS syndromic spectrum. This study approach provides a unique view of the shared clinical and genetic architecture that influences both PCOS as a discrete diagnosis and the symptoms of PCOS that affect a large number of females.

## Data Availability

Restrictions apply to the availability of data generated or analyzed during this study to preserve patient confidentiality or because they were used under license. The corresponding author will on request detail the restrictions and any conditions under which access to some data may be provided.

## Abbreviations

BMI: body mass index
EHR: electronic health record
GWAS: genome-wide association study
ICD: International Classification of Diseases
NIH: National Institutes of Health
OR: odds ratio
PCOS: polycystic ovary syndrome
PPV: positive predictive value
PR: polygenic risk score
SNV: single-nucleotide variation
VUMC: Vanderbilt University Medical Center

## Additional Information

***Disclosure Summary:*** The authors have nothing to disclose.
